# Isolation and Characterization of AbTJ, an *Acinetobacter baumannii* Phage, and Functional Identification of Its Receptor-Binding Modules

**DOI:** 10.3390/v12020205

**Published:** 2020-02-12

**Authors:** Jingzhi Xu, Xiaobo Li, Guangbo Kang, Liang Bai, Ping Wang, He Huang

**Affiliations:** 1 Department of Biochemical Engineering, School of Chemical Engineering & Technology, Tianjin University, Tianjin 300350, China; 2017207003@tju.edu.cn (J.X.); li_xiaobo@tju.edu.cn (X.L.); 1017207102@tju.edu.cn (G.K.); bailiang20101@163.com (L.B.); 2 Key Laboratory of Systems Bioengineering (Ministry of Education), Tianjin University, Tianjin 300072, China; 3 Tianjin Modern Innovative TCM Technology Co. Ltd., Tianjin 300392, China

**Keywords:** *A. baumannii*, phage AbTJ, bioinformatics analysis, tail fiber protein (TFP), fluorescent and bioluminescent methods

## Abstract

*A. baumannii* is an opportunistic pathogen and a major cause of various community-acquired infections. Strains of this species can be resistant to multiple antimicrobial agents, leaving limited therapeutic options, also lacking in methods for accurate and prompt diagnosis. In this context, AbTJ, a novel phage that infects *A. baumannii* MDR-TJ, was isolated and characterized, together with its two tail fiber proteins. Morphological analysis revealed that it belongs to *Podoviridae* family. Its host range, growth characteristics, stability under various conditions, and genomic sequence, were systematically investigated. Bioinformatic analysis showed that AbTJ consists of a circular, double-stranded 42670-bp DNA molecule which contains 62 putative open reading frames (ORFs). Genome comparison revealed that the phage AbTJ is related to the *Acinetobacter* phage Ab105-1phi (No. KT588074). Tail fiber protein (TFPs) gp52 and gp53 were then identified and confirmed as species-specific proteins. By using a combination of bioluminescent methods and magnetic beads, these TFPs exhibit excellent specificity to detect *A. baumannii*. The findings of this study can be used to help control opportunistic infections and to provide pathogen-binding modules for further construction of engineered bacteria of diagnosis and treatment.

## 1. Introduction

*Acinetobacter baumannii (A.baumannii)*, a non-fermentative, Gram-negative bacteria found in water, soil, sewage and many healthcare environments, is a common opportunistic pathogen that can cause severe hospital infections in the skin, urinary system, and other tissues and systems [1]. Infections due to these bacteria, especially carbapenem-resistant strains of *A. baumannii*, are associated with high mortality. With the long-term application of a large number of broad-spectrum antibacterial drugs, the problem of antibiotic resistance gets worse every year. Clinical *A. baumannii* strains are developing multi- (MDR) and Pan-drug resistance (PDR) that imperils the effectiveness of medical treatments for many infectious diseases, even in the most developed countries [2,3]. Specific detection of pathogenic bacteria is the key to treating these threats and there needs to be a clinical focus on finding novel detection methods. Conventional diagnostic methods require time-consuming bacterial culture and labor-intensive DNA extraction [4] and alternative efforts for rapidly detecting bacteria need to be developed. Phages are considered to be useful diagnostic tools with the potential to treat bacterial infections [5,6] and over the past decade, more attention has been paid to phage therapy once antibiotics fail to treat a disease. They also cause minimal harm to the human body and there is therefore a growing demand for new phages to be isolated and characterized [7].

Phages are extremely abundant bacterial parasites which can recognize, capture, and lyse their specific host bacteria in a variety of harsh environments. Research has shown that phages possess the ability to control and regulate the structure of the microbial community and play a vital role in biogeochemical cycles [8,9]. During the phage life cycle, host bacteria are captured by the tail fibers of the phage. Tail spikes then dissolve the bacterial cell wall to allow the phage’s nucleic acid to penetrate the bacteria and progenies are then assembled using the host’s replication machinery. Finally, endolysin accumulation in the endopeptidase glycan layer causes host lysis and the release of mature phage offspring [4,10]. Although it has been reported that intact phages can detect bacteria with a high degree of specificity, their inherent strong lytic activity against host bacteria hinders downstream identification and detection [11,12]. The tail fiber protein (TFP) is responsible for the initial recognition of the specific host bacteria and therefore it might be used as a potential bio-recognition element to detect bacteria. Information gained from the studies of the interaction between the receptor-binding proteins and the bacterial host receptor will provide a foundation for using phage to detect, prevent, and control bacterial infections.

The whole genome sequence of strain MDR-TJ was determined in our previous work, which was found to be resistant to penicillin, cephalosporins, aminoglycosides, quinolones, and also imipenem [13,14]. Research showed that *A. baumannii* MDR-TJ harbored a genomic resistance island that can interrupt the comM gene and a putatively conjugative plasmid, pABTJ1, which mediated MDR-TJ’s resistance to carbapenem [13]. Besides, the remaining large scaffold of *A. baumannii* MDR-TJ, a new plasmid pABTJ2, was further finalized which carries many phage-like elements [15]. Studies on *A. baumannii* MDR-TJ are currently driving research on bacterial viruses, in an effort to implement phage therapy and phage-derived antimicrobials. Recently, in our laboratory, we screened and isolated AbTJ, an *A. baumannii* phage against MDR-TJ, from hospital sewage. Its biological characteristics were observed and its complete genome sequence was analyzed. For this phage, gp52 and gp53 were predicted to be the genes for the TFPs which might be responsible for initial *A. baumannii* recognition. We also investigated the TFPs capability to act as a non-lytic, specific, and stable biorecognition element for *A. baumannii*. Using a method that combined magnetic beads and bioluminescence, we successfully detected *A. baumannii* in biological samples by calculating the bacterial recovery rate. This study not only deepens our understanding of AbTJ, but is also important for the development of phage treatments for diseases caused by *A. baumannii*.

## 2. Materials and Methods

### 2.1. Bacterial Strains and Growth Conditions

The *A. baumannii* strain MDR-TJ was isolated from a sputum sample from the Second Hospital of Tianjin Medical University (Tianjin, China), a tertiary-care comprehensive university hospital. The whole genome sequence of the *A. baumannii* strain MDR-TJ used was previously determined by our group. The sequences were deposited into GenBank under the accession number NC_017847. Bacteria used in this experiment were cultured in lysogeny broth at 37 °C/200 rpm and bacterial cultures were stored at −80 °C in 30% glycerol.

### 2.2. Source and Isolation of Phages

Sewage samples were clarified by centrifugation at 5000× *g* for 20 min, passed through a 0.22 μm filter, and stored at 4 °C until required. We used the MDR-TJ isolated above as a host strain for the phages according to the isolation technique recommended by Luo Juan [16,17] with some modifications. In brief, after the sewage filtrate and LB medium were mixed in an equal volume, 2 mL of *A. baumannii* host bacteria solution (OD(600) = 0.6) was added to the mixture, incubated at 37 °C with shaking overnight to enrich *A. baumannii*-specific phages. The supernatant was collected by centrifugation the next day and filtered through a 0.22 μm filter to remove bacterial cells and debris. The filtrate after filtration is the enriched *A. baumannii* phage solution. To confirm phage presence in the filtrate, the double-layer agar plate method was used to obtain single-plaque isolations which were collected and stored in 1 mL of SM buffer at 4 °C. This procedure was repeated at least three times to obtain pure phage. The presence or absence of plaque formation indicated the susceptibility of *A. baumannii* MDR-TJ strains to isolated phages. Isolated phages were further purified by precipitation with polyethylene glycol (PEG). Plaque assays to determine the number of plaque-forming units (PFUs) in the phage solutions were performed using the double-layer method.

### 2.3. Transmission Electron Microscopy

Phage particles were adsorbed onto a freshly prepared carbon-coated copper grid and negatively stained with 2% phosphotungstic acid (pH 7.0). Purified phage morphology was examined using a Hitachi transmission electron microscope H-9500 (Tokyo, Japan) operating at 100kV according to the method described by Mendes et al. [18].

### 2.4. One-Step Growth Curve and Host Range Analysis

The multiplicity of infection (MOI) was measured by the gradient dilution and double-layer agar method [19]. The one-step phage growth curve can be divided into three periods: incubation, lysis, and stationary. Phages were added at an MOI of 0.01. For one-step growth curve experiments a modification of the method of Luo et al. [16] was used, with 15 min adsorption at 37 °C, then centrifugation at 13,000 rpm for 30 s to remove any unabsorbed phage particles. The pelleted cells were resuspended in 5 mL of preheated (37 °C) LB broth and incubated at 37 °C. Samples were taken at 10-min intervals up to 180 min and PFU titers were immediately obtained. Experiments were repeated at least three times with duplicate samples. The phage’s host range was investigated using 21 different bacterial strains stored at our laboratory, determined by standard spot assay, and confirmed by the double-layer agar method. A blank culture was used as a control and this experiment was conducted in triplicate.

### 2.5. Stability Studies

A temperature-controlled incubator or water bath was used to determine the phage stability at a range of pHs, temperatures, disinfectants, and ions [20]. First, the titer of the original phage in this experiment was determined. Five temperatures (37 °C, 50 °C, 60 °C, 70 °C, 80 °C) were selected to study AbTJ’s thermal tolerance in LB broth for 60 min with samples taken every 10 min. To examine pH-stability, phage samples were incubated for 2 h at 37 °C in a range of pHs (adjusted using NaOH or HCl) from 2 to 12. To examine phage sensitivity to disinfectants, biological resistance was determined using the conventional biocides ethanol (10%, 50%, 75%, and 95% *v/v*) and isopropanol (10%, 50%, and 95%) at 15 min intervals for sampling. Finally, to examine the influence of Ca^2+^ and Mg^2+^ on phage lysis, phage-infected bacteria were incubated on LB agar with and without CaCl_2_ or MgCl_2_ at a range of concentrations (0, 5, 10, 15, 20, 25, and 30 mmol/L). Bacteriophage titers were all determined using the double-layer agar plate method incubated at 37 °C for 12 h.

### 2.6. Phage DNA Extraction and Genome Sequencing

Phage DNA extraction was performed using the method of Bo Changqing [21]. Purified phage genomic DNA was sequenced using Illumina Hiseq 2000 platform by Benagen Biotech Company (Wuhan, China) and a custom 2X150 bp paired-end DNA library was prepared with an average fragment length of 350 bp. All raw Illumina sequence data were quality filtered with SOAPnuke (version: 1.3.0), a sequence filtering program developed at BJI, used to remove reads with adapters containing more than 5% N and low-quality reads (quality value less than or equal to 5) [22,23]. De novo assembly was performed with SPAdes (version: 3.10.1; parameter: −127) [24], which can match sequencing data from multiple platforms. The assembly was circular with a relatively homogenous coverage. BWA (version: 0.7.12-r1039) was used to match the clean data to all assembled sequences and SOAP. coverage (version: v2.7.7), which can compute the average depth and GC content of each window and make the GC-depth map, was used to calculate the depth of coverage at each site. Based on the clean data, we used the appropriate data volume to assemble and process subsequent analysis with the purpose of obtaining the complete genome sequence.

### 2.7. Whole-Genome Bioinformatic Analysis

The full length (42670bp) of the phage genome was analyzed using EditSeq software in the DNAStar package. The analysis included; A, T, G, and C base composition, G + C content, the average length of calculated genes, and the density of genes in the genome [5]. Open reading frame (ORF) prediction was carried out with the NCBI ORF finder (https://www.ncbi.nlm.nih.gov/orffinder), GeneMark.hnm (http://topaz.gatech.edu/GeneMark/gmhmmp.cgi), and Softberry (http://linux1.softberry.com/berry.phtml?topic=virus&group=programs&subgroup=gfindv). Basic Local Alignment Search Tool (BLAST) (http://www.ncbi.nlm.nih.gov/BLAST/) was used to identify putative proteins sharing similarities with the predicted phage proteins through the Blastp program, and select the results with an E value less than e-5 based on the actual situation to define the function of the gene. Putative tRNA-encoding genes were analyzed using tRNAscan-SE 1.21 (http://lowelab.ucsc.edu/tRNAscan-SE/) [6,25]. We used the CpG islands function from the European bioinformatics institute of EMBL-EBI website (http://www.ebi.ac.uk/emboss/cpgplot), and the CpGFinder PROMOTER softberry website options (finder GC-islands) function (http://www.softberry.com/berry.phtml) to analyze the distribution of phage genome GC. A phage genome map was presented using DNA plotter [26,27]. The CLC Main Workbench, version 8 was used for genome annotation [28]. Phylogenetic analysis with other related phages was carried out by comparing the whole gene sequences of the other Acinetobacter phages, and the phylogenetic trees based on the neighbor-joining algorithm were conducted by using the genetic analysis software MEGA 6. Whole-genome comparisons were carried out using Mauve software [29,30].

### 2.8. Expression, Purification, and Preparation of FITC-Labelled TFPs

An *E. coli* expression system was used to produce the TFPs gp52 and gp53. A single positive clone included preparation of phage template DNA, PCR amplification of TFP gene, construction of recombinant pET32a-gp52 and pET32a-gp53 gene, TFPs expression, and purification. In brief, the recombinant plasmid of pET32a-gp52/gp53 was transformed into *E. coli* Trans B for screening the positive clones. Single positive clone was cultured in LB medium containing ampicillin at 37 °C and shaken continuously at 180 rpm until OD600 reached 0.6. Then the bacterial culture was added 10 mM isopropyl thiogalactoside (IPTG), followed by overnight culture at 16 °C. Finally, the bacterial solution was disrupted after collection and using the AKTA protein purification instrument (General Electric Company, Boston, MA, USA) to purify the TFPs. After purified TFPs were produced, they were labeled with FITC (fluorescein isothiocyanate) [4]. One milliliter of FITC dissolved in DMSO (3.0 mg mL^−1^) was mixed with 1.0 mL of TFPs solution at 0.5 mg mL^−1^. After the mixture incubation for 12 h at 4 °C, the reaction was stopped by adding 200 μL of 500 mM NH_4_Cl. The solution was dialyzed against 10 mM PBS for 48 h at 4 °C.

### 2.9. Immunofluorescence Imaging of FITC-Stained A. baumannii

One hundred microliters of *A. baumannii* MDR-TJ suspension at 1.5 × 10^8^ CFU mL^−1^ was mixed with 300 μL of FITC-labelled TFPs, followed by 90 min incubation at 37 °C. After thorough centrifuging and washing, the stained *A. baumannii* was resuspended in 10 mM PBS. Then 10 μL of the specimen was dropped onto a glass slide and covered by a cover glass; it was observed under the FL microscope.

### 2.10. Bioluminescent and Fluorescent Methods for Functional Identification Using TFPs

For the BL method, 100 μL of TFPs solution at 100 μg mL^−1^ together with coating solution (0.05 mol/L sodium carbonate-sodium bicarbonate buffer, pH 9.6) were added onto a 96-well microplate and incubated at 37 °C for 2 h. The coated microplate was blocked with 150 μL of blocking solution for 2 h at 37 °C after washing with PBST. Then 100 μL of 1.5 × 10^7^ CFU mL^−1^ *A. baumannii* suspension was added onto the microplate and incubated for 1 h at 37 °C. After washing three times with PBST, the same volume of blank solvent, TFPs solution and 1.0 × 10^7^ CFU mL^−1^ phage suspension were added onto the microplate above, respectively, to verify the lysis of TFPs by detecting the ATP signal value according to the instructions of the ATP kit with microplate fluorometer and luminometer instrument. For the sandwich FL method, 100 μL of TFPs solution at 100 μg mL^−1^ together with coating solution were added onto a 96-well microplate and incubated at 37 °C for 2 h. The coated microplate was incubated with 150 μL of blocking solution for 2 h at 37 °C. Then 100 μL of *A. baumannii* suspension was added into the microplate for incubation at 37 °C for 1 h. After washing three times with PBST, 100 μL of 100 μg mL^−1^ FITC-labelled TFP solution was added to the microplate and incubated for 30 min at 37 °C. The FL signal on the microplate was detected with an excitation wavelength of 544 nm and an emission wavelength of 580 nm on the microplate fluorometer and luminometer instrument [4]. TFPs specificity was evaluated by the interference degree (ID) values of the interfering bacteria, calculated by the following equation.

ID = (*I − B*)/(*A − B*) × 100% (1)

Here *I*, *A*, and *B* represent the FL signals from the interfering bacteria, *A. baumannii* MDR-TJ and a blank sample, respectively [4].

### 2.11. TFPs-MBs Preparation and Use in A. baumannii Detection

100 μL of 2 μm NHS magnetic beads (MBs) was washed with PBS (pH8.5) at least three times and then added to a mixture containing 100 μL of activation buffer and 200 μL of 0.25 mg mL^−1^ TFP, and incubated for 6 h at 25 °C with gentle shaking to activate the beads. To block the activation sites of unconjugated proteins, the beads were incubated for 2 h at 25 °C with shaking in a solution containing 10% skim milk buffer. The blocked TFPs-MBs were stored in 0.1% sodium azide PBST solution buffer after thoroughly washing them. For magnetic beads combined with BL method for *A. baumannii* detection, 1 mL different concentrations of an *A. baumannii* suspension was incubated with 20 μL of TFPs-MPs for 60 min at 37 °C. Bacteria were detected by the ATP signal value according to the instructions of the ATP kit with the instrument of microplate fluorometer and luminometer.

### 2.12. Nucleotide Sequence Accession Number

The complete genome sequence of the *A. baumannii* phage AbTJ is accessible in the GenBank database [28] under the accession number MK340941.

## 3. Results

### 3.1. Isolation and Morphology of A. baumannii Phages

**Figure 1:**
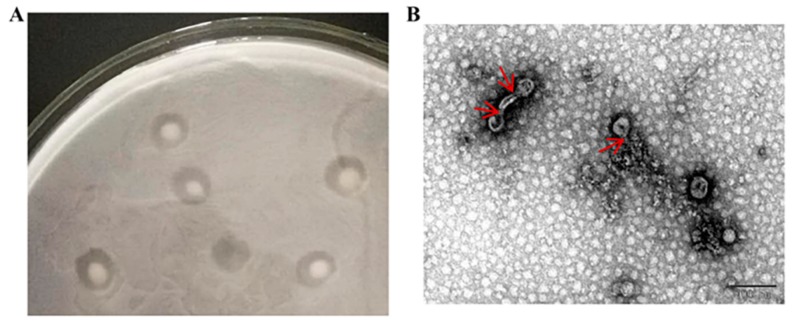
Plaque and TEM morphology of phages. (**A**) AbTJ plaque morphologies. (**B**) TEM morphology of phage AbTJ. Phages were negatively stained with potassium phosphotungstate. Scale bar, 100 nm. The red arrows point to the phage’s tails.

Phages were isolated from sewage with *A. baumannii* MDR-TJ. Purified and concentrated phage solutions were observed using a transmission electron microscope (Figure 1). The plaques have various shapes, some form halo circles, some are multiple concentric circles, and some are approximately circular; transparent plaques are typically lytic phages, and turbid plaques of different sizes may be characteristic of temperate phages. As shown in Figure 1, the large plaques of AbTJ were about 3–6 mm in diameter and contained a turbid zone on the top agar (Figure 1A), and AbTJ has an icosahedral head (approximately 55 nm in diameter) and contractile tail (approximately 15 nm in length) (Figure 1B). These morphological features suggest that the phage AbTJ belongs to the *Podoviridae* virus family.

### 3.2. One-Step Growth Curve and Host range

**Figure 2:**
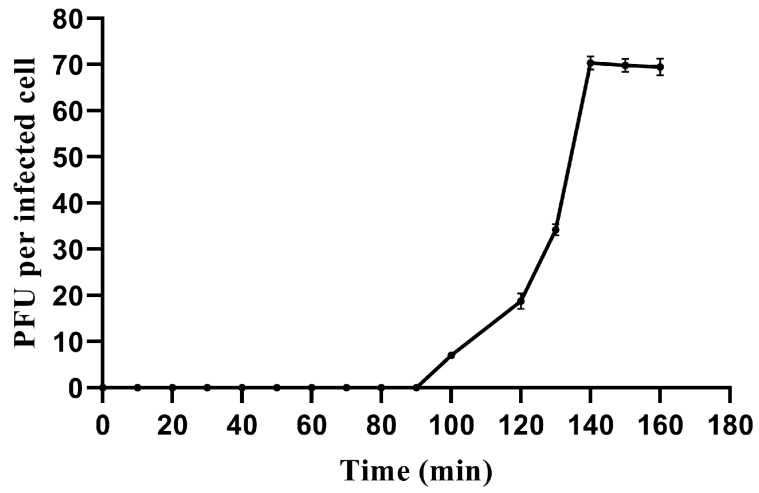
One-step growth curve of AbTJ. Phages were grown in an exponential phase culture of *A. baumannii* MDR-TJ. Graph shows PFU per infected cell in cultures at different time points. Each data point is the mean from three experiments.

We used a one-step growth curve experiment to determine its latent period and phage burst size, characteristics which are closely related and influenced by many factors. The one-step growth curve showed that AbTJ had a latent period of 90 min and a rise period of 50 min. The plateau phase was reached at 140 min when AbTJ produced the maximum number of progeny (1 × 10^7^ PFU/mL). The average burst size of the phage AbTJ was 70 pfu/cell (burst size is the number of phages produced/infected bacterium) (Figure 2). Host range tests suggested that among all the species tested, AbTJ was specifically virulent to only five *A. baumannii* strains (Table 1). In the table above, all *A. baumannii* strains except ATCC19606 were shown to be resistant to aminoglycoside and ciprofloxacin. However, AbTJ did not infect the rest of the strains, suggesting that it does not possess a broad host range.

### 3.3. Phage Stability Tests

**Figure 3:**
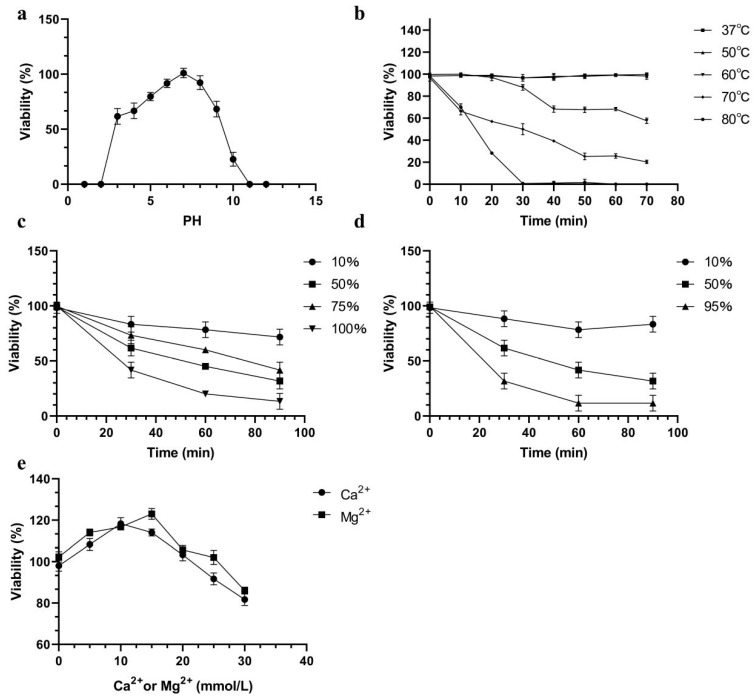
AbTJ resistance to physical and chemical agents. (**a**) Effect of pH on phage titer after incubation for 60 min in LB broth at 37 °C. (**b**) Inactivation kinetics of AbTJ at 37 °C, 50 °C, 60 °C, 70 °C, and 80 °C. (**c**) Inactivation kinetics of ABTJ in the presence of 10%, 50%, 75%, and 100% ethanol. (**d**) Inactivation kinetics of ABTJ in the presence of 10%, 50%, and 95% isopropanol. (**e**) Effect on AbTJ titer of incubation in LB broth with and without Ca^2+^ or Mg^2+^ (0, 5, 10, 15, 20, 25, and 30 mmol/L) at 37 °C. For all graphs, values represent the mean of three experiments.

The pH sensitivity of AbTJ is shown in Figure 3a. The phage can remain stable over a broad pH range from 3 to 10 at 37 °C. It was noticed that still nearly half of the phages survived at pH 3 and 9. The phage titer decreased by varying amounts at a pH above or below 7. At pH 10, reductions of 81.25% in phage particle counts were observed. Few viral particles were detected at pH 2 and pH 11. The viability loss when AbTJ was incubated at different temperatures is shown in Figure 3b. The phage titer reduced rapidly at 60 °C, 70 °C, and 80 °C and was completely inactivated when incubated at 80 °C for 35 min. No reduction in the phage titer was observed at 37 °C or 50 °C after 75 min incubation. The survivor curves for AbTJ were affected by the presence of ethanol and isopropanol (Figure 3c,d). The most effective concentrations of ethanol (100%, *v/v*) and isopropanol (95%, *v/v*) reduced the phage titer after 90 min by 82% and 91%, respectively. Because many phages require divalent ions such as Ca^2+^ or Mg^2+^ for optimal adsorption, the ion-dependence of AbTJ was determined. The most efficient infection was achieved with concentrations of 15 mM Mg^2+^ (125%) and 10 mM Ca^2+^ (120%) (Figure 3e).

### 3.4. Whole-Genome Analysis of Acinetobacter Phage AbTJ

**Figure 4:**
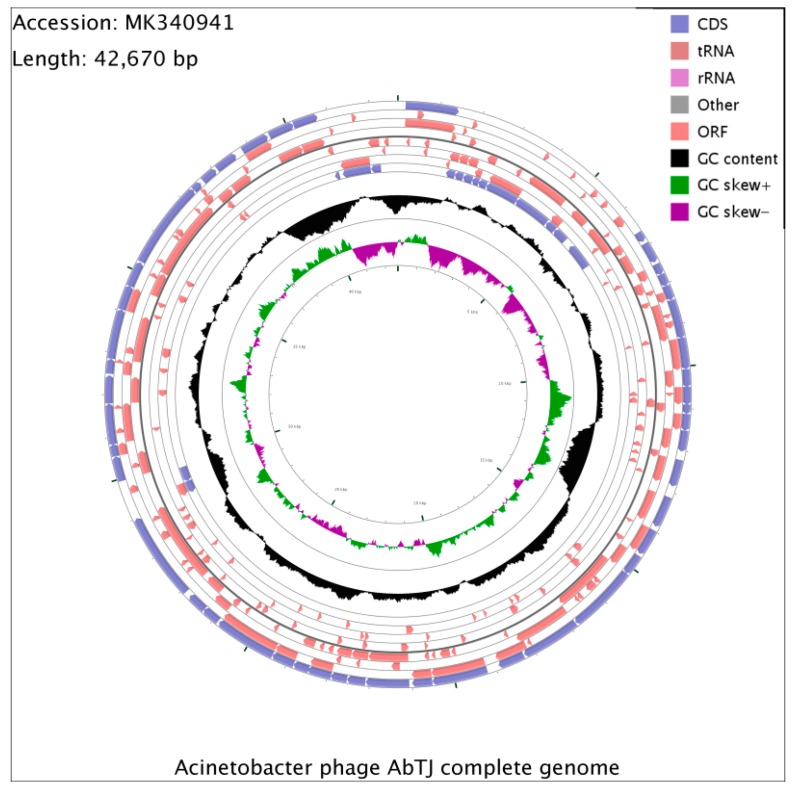
A circular representation of the phage AbTJ genome. Circles display (from the outside): (1) physical map scaled in kb; and (2) Coding sequence (CDS) in blue. (3) Open reading frames (ORFs) transcribed in the clockwise or the counterclockwise direction. ORF coding functional proteins are in magenta. (4) G+C % content (in a 1-kb window and 0.1-kb incremental shift) in black. (5) GC skew (G – C/G + C, in a 1-kb window and 0.1-kb incremental shift). Values greater than zero are in green and the smaller are in purple.

**Figure 5:**
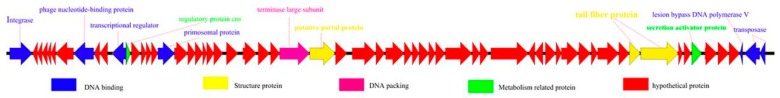
Genomic map of phage AbTJ. AbTJ genomic map was constructed with CLC Main Workbench, version 8. The AbTJ genome is schematically presented with the predicted ORFs indicated by arrows with arrow direction representing transcription direction. Different colors denote different functional groups of phage genes.

**Figure 6:**
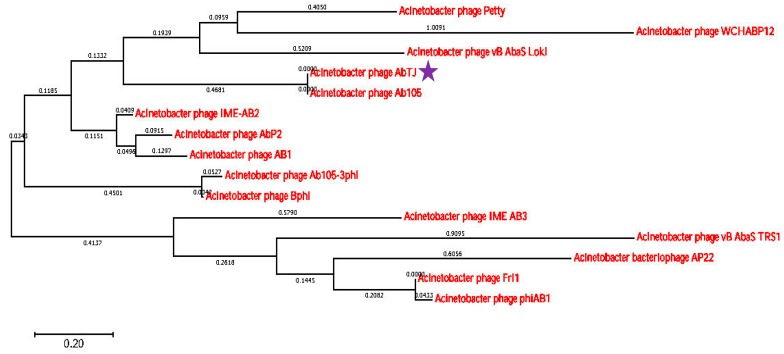
Phylogenetic tree based on whole genomes of selected bacteriophages. Whole genomes were compared using ClustalW and the phylogenetic tree generated using the neighbor-joining method and 0.20 bootstrap replicates. The star represents the phage screened in this experiment.

**Figure 7:**
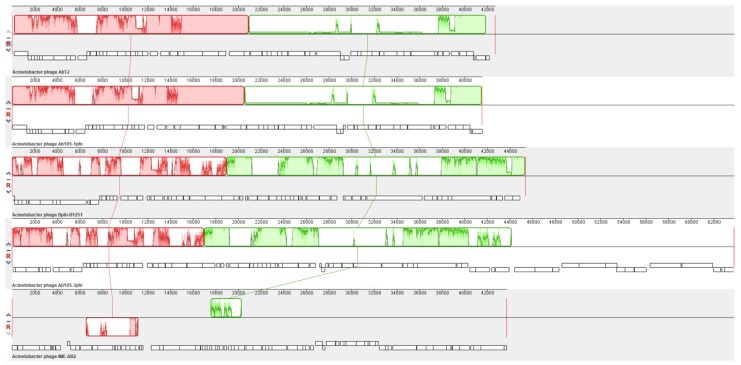
Multiple genome alignment generated by Mauve software (http://asap.ahabs.wisc.edu/mauve/). The chromosomes of Acinetobacter phages AbTJ, IME AB2, Ab105-3phi, Ab105-1phi, and Bphi-B1251 were compared. Genome similarity is represented by bar height, which corresponds to the average conservative level in this region of the sequence. A completely white region indicates a fragment that is not aligned or contains a particular genome-specific sequence element. Boxes with identical colors represent local colinear blocks (LCB), indicating homologous DNA regions shared by two or more chromosomes without sequence rearrangements.

The whole phage genome was sequenced and found to consist of a circular double-stranded 42,670 bp DNA molecule, with a G+C content of 39.32% and no detected tRNA. Analysis of the sequence found that the restriction endonuclease SacII and DrdI had one cutting site respectively in the genomic DNA. Thus, it was expected that when SacII and DrdI were together used to digest the DNA, three fragments would be generated if the DNA comprised a linear genome, but if the genome was circular, two fragments would be generated. Experiments were also conducted using both endonucleases alone. The digestion experiment generated two fragments using two endonucleases (Supplementary Figure S1A) and one fragment using one endonuclease (Supplementary Figure S1B) respectively in the agarose gel, revealing that the AbTJ genome is a circular molecule. A total of 88 ORFs larger than 150 bp were found using ORF finder, and 62 putative genes were identified among these ORFs, with an average length of 643 bp. The entire genome structure of AbTJ is shown in Figure 4 and putative functional assignments and significant similarities to other sequences are listed in Table 2. The orientation of genome annotation was chosen so that most genes (47; 76%) were located on the positive strand while only 15 genes (24%) were located on the negative strand. BLAST analysis of the complete genome sequence search on the GenBank database showed that 13 putative proteins exhibited significant similarity to the viral proteins in the non-redundant protein sequences database while the remaining 49 ORFs were not found to match any functional proteins. As shown on the genome map (Figure 5); the arrows represent the predicted ORFs consisting of genes involved in the bacteriophage structure and DNA replication as well as other predicted functions. Based on our analysis, the annotated proteins of AbTJ can be categorized into the following functional groups: DNA binding (integrase; nucleotide-binding protein, transcriptional regulator, primosomal protein, DNA polymerase V, transposase), structural proteins (portal protein, tail fiber protein), DNA packing (large terminase subunit), metabolism-related proteins (regulatory protein cro, secretion activator protein), and hypothetical proteins (Figure 5). More detailed in-depth studies are needed to fully understand the nature of this novel phage. No virulence or antibiotic resistance genes were found in the genome after searches using the VFDB and ARDB databases, which implies in vivo safety of the phage. To investigate AbTJ’s phylogeny, phylogenetic analysis based on complete genomic comparisons was performed using neighbor-joining analysis, and a tree was constructed using MEGA 6. As shown in Figure 6, AbTJ was most closely related to the phage Ab105- 1phi (No. KT588074.1). A multiple genome alignment of the chromosomes of phages AbTJ, IME AB2, Ab105-3phi, Ab105-1phi, and Bphi-B1251 confirmed their relatedness and indicated that AbTJ shared a high sequence similarity (>90%) with the Acinetobacter phage Ab105-1phi, which belongs to the *Podoviridae* family, as the electron micrograph above confirmed. The phages had a very similar genome size of approximately 41–43 kb (Figure 7). The annotated genome sequence of AbTJ should contribute to a better understanding of the classification of these related phages.

### 3.5. Identification the Function of the Phage Tail Fiber Protein (TFP)

**Figure 8:**
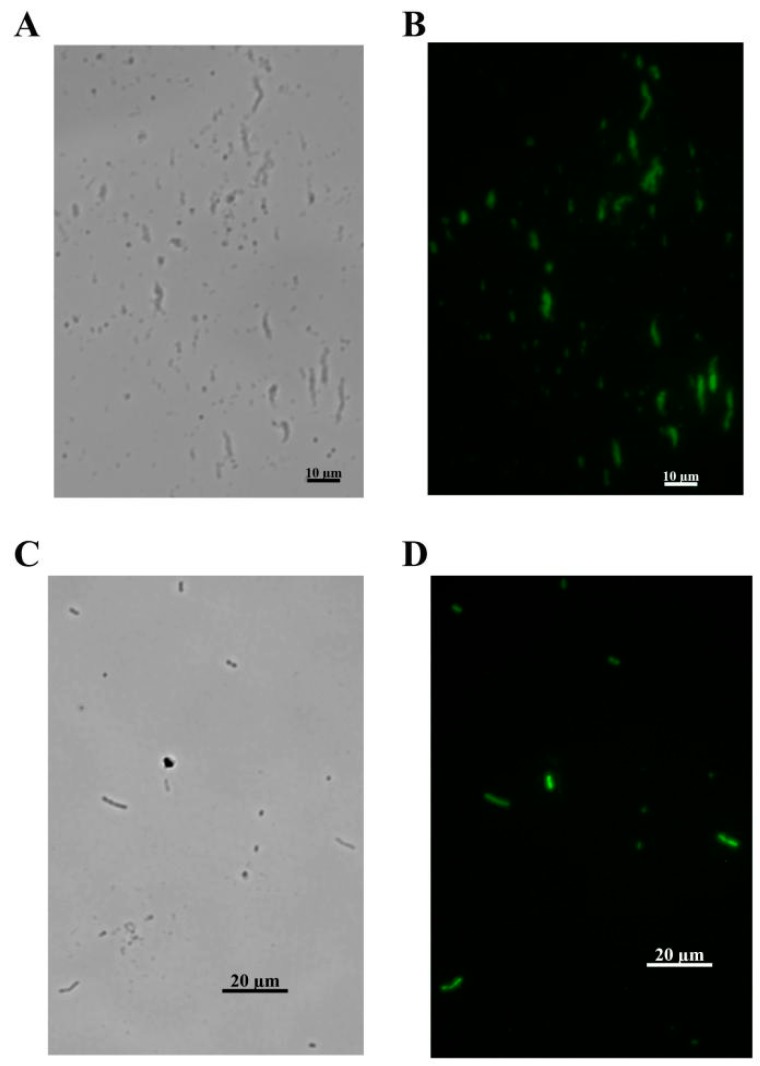
FL micrographs of the stained *A. baumannii*. (**A**) The green FL channel of FITC-labelled gp52 stained bacterium. (**B**) The bright field of FITC-labelled gp52 stained bacterium. (**C**) The green FL channel of FITC-labelled gp53 stained bacterium. (**D**) The bright field of FITC-labelled gp53 stained bacterium.

**Figure 9:**
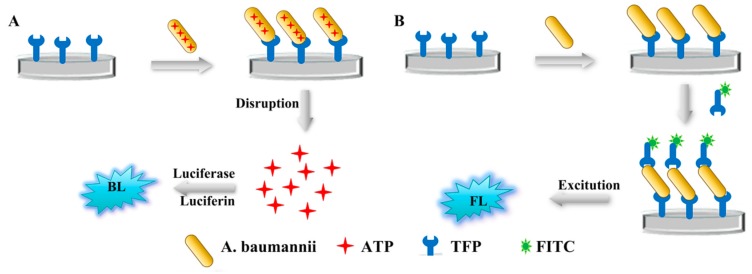
Schematic illustration of (**A**) one-site BL method and (**B**) sandwich FL method.

**Figure 10:**
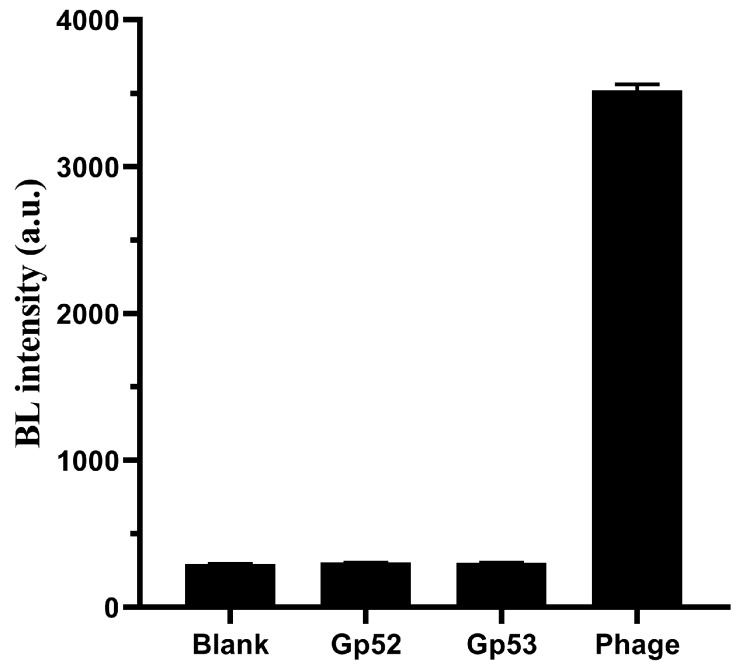
BL signals of 200 μL of 1.5 × 10^7^ CFU mL^−1^ *A. baumannii* suspension treated with PBS buffer, 1.0 × 10^7^ PFU mL^−1^ phage suspension and 100 μg mL^−1^ gp52 and gp53 solution (*n* = 4).

**Figure 11:**
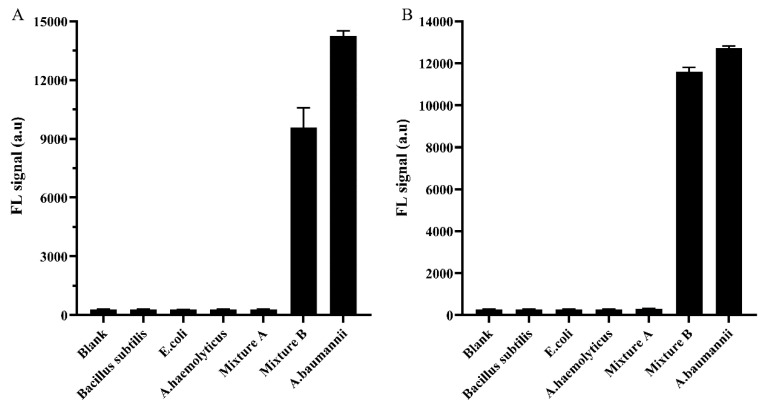
TFPs specificity. The specificity of gp52 (**A**) and gp53 (**B**). The concentration of all tested bacteria was 1.5 × 10^7^ CFU mL^−1^ (*n* = 4).

**Figure 12:**
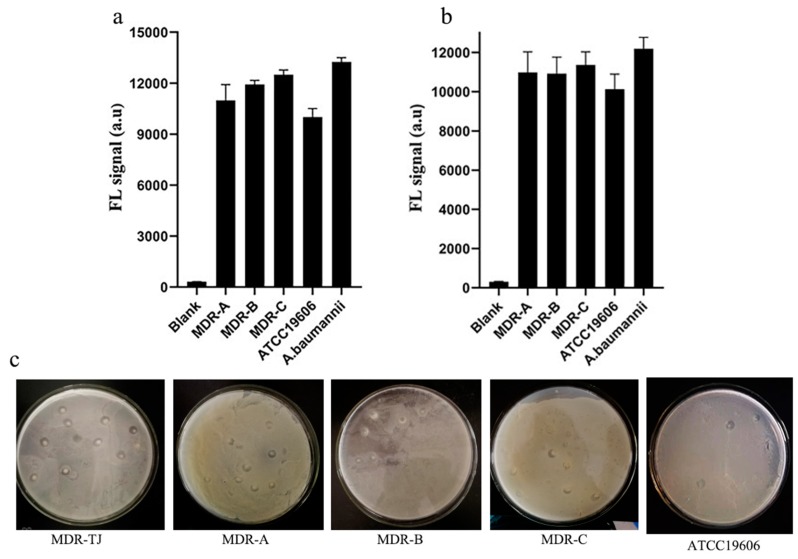
Strain specificity of gp52 (**a**) and gp53 (**b**). Photographs showing plaques of the phage infecting five strains of *A. baumannii* (**c**). The concentration of all tested bacteria was 1.5 × 10^7^ CFU mL^−1^ (*n* = 4).

The adsorption of the phage on the surface of bacteria is the first and most important step in the process of phage infection [31]. In this study, the genes *gp52* (No. MN524652) and *gp53* (No. MN524653) were predicted to express TFPs likely to be involved in the initial recognition of *A. baumannii* MDR-TJ. The molecular weight (MW) of the obtained recombinant protein is the same as the theoretical estimated MW of gp52 and gp53. The binding activity of tail fiber protein was investigated by visualizing *A. baumannii* MDR-TJ interactions with fluorescein isothiocyanate (FITC)-labeled gp52 and gp53. Observation of FITC on *A. baumannii* cells under the fluorescence microscope (Figure 8) indicated that the tail fiber proteins gp52 and gp53 had the ability to recognize and capture *A. baumannii*. Since they are an initial recognition element, TFPs should lack the lytic activity of intact phage. The lytic activity of TFPs was evaluated by detection of bioluminescence signals (Figure 9A) produced by intracellular ATP release. *A. baumannii* MDR-TJ suspensions were mixed with either TFP solution or phage suspension. The bioluminescence signals from the phage-containing mixtures were more than 15 times greater than those seen in the TFP mixtures (Figure 10). These results indicate that TFPs lack the lytic activity of intact phage, and can be used for identification and detection. The specificity of TFPs using the sandwich FL method (Figure 9B) was evaluated by studying their binding activity to two Gram-native bacteria (*A. haemolyticus* and *E. coli*), a Gram-positive bacteria (*Bacillus subtilis*), and four other *A. baumannii* strains. As shown in Figure 11A,B, the ID values of the three interfering bacteria and mixture A (all three interfering bacteria) were all below 2%. However, the ID values of the Mixture B, prepared by mixing the above-mentioned interfering bacteria with *A. baumannii* MDR-TJ, were 91.2% and 93.7%, respectively. Strain specificity of TFPs are shown in Figure 12. The fluorescence signals from MDR-A, MDR-B, MDR-C, and ATCC19606 showed minor differences in comparison with those from MDR-TJ (Figure 12a,b). The phage lysed all strains above and formed transparent plaques (Figure 12c). These results demonstrate that TFPs cannot recognize pathogens from other generas but can distinguish between different strains, which greatly enhances their potential use in clinical applications.

#### Baumannii Detection Using TFPs and TFPs Use in Diverse Samples

**Figure 13:**
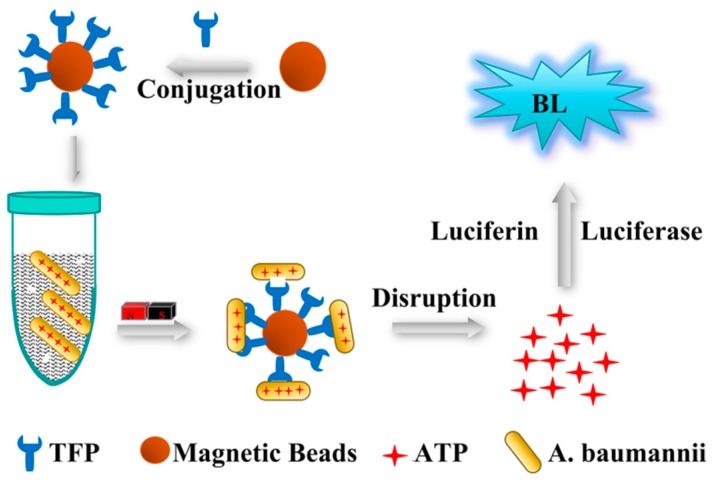
Schematic illustration of TFPs recognition-based magnetic beads combined with one-site BL method for *A. baumannii* detection.

**Figure 14:**
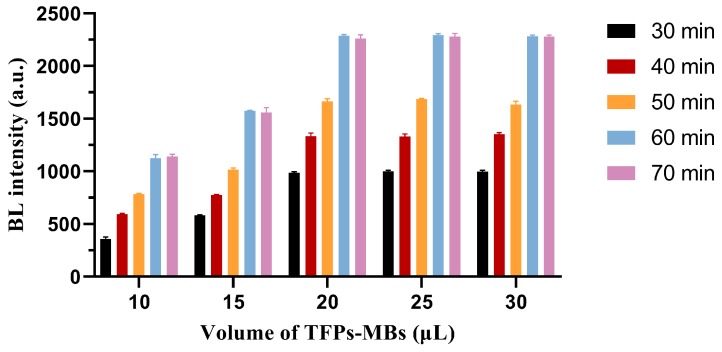
Effects of volume of TFPs-MBs and incubation time on BL responses to *A. baumannii* at 1.5 × 10^7^ CFU mL^−1^ (*n* = 4).

**Figure 15:**
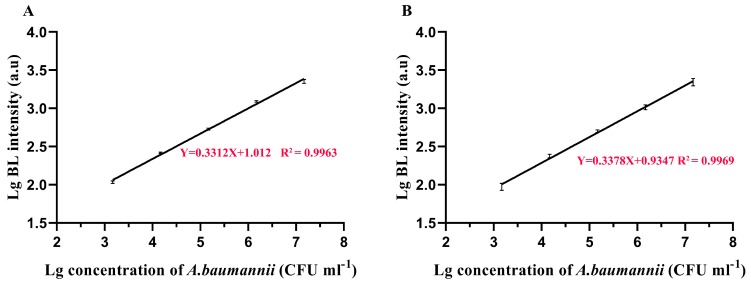
Performance for *A. baumannii* detection using TFPs-MBs with BL method by gp52 (**A**) and gp53 (**B**). All the detection conditions were optimal conditions (*n* = 4).

A method of combining magnetic beads with one-site BL was established for *A. baumannii* detection using these TFPs as the biorecognition element (Figure 13). BL scans with different dilution factors showed that the BL value decreased linearly with bacterial concentration. With the optimized TFPs-MBs volume and *A. baumannii* incubation time of 20 μL and 60 min respectively (Figure 14), the detection limits of gp52 and gp53 were 6.2 × 10^2^ CFU mL^−1^. The gp52 regression equation was lg I (a.u.) = 1.012 + 0.3312 × lg C (CFU mL^−1^), with a correlation coefficient of 0.9963 (Figure 15A). The gp53 regression equation was lg I (a.u.) = 0.9347 + 0.3378 × lg C (CFU mL^−1^), with a correlation coefficient of 0.9969 (Figure 15B). Here C and I represent the bacterial concentration and the BL intensity, respectively. This method detects stable signals and is highly sensitive. In summary, the tail fiber proteins gp52 and gp53 have broad clinical detection application prospects as a molecular recognition reagent for *A. baumannii*. The above method was also used to evaluate the potential of TFPs to detect *A. baumannii* in clinical samples. Varying *A. baumannii* MDR-TJ concentrations were added to glucose (10%), human urine, feces, sputum, and domestic sewage, and the concentration of captured bacteria was detected. The use of TFPs in the detection of *A. baumannii* MDR-TJ was evaluated by the recovery rate of the tested bacteria. As shown in Table 3 and Table 4, the recovery of test samples is all above 50%, with relative standard deviation (RSD) values all below 6%. These results indicate that the TFPs could be used as bio-recognition elements for detecting *A. baumannii* in complicated samples with acceptable reliability.

## 4. Discussion and Conclusions

As a “Priority 1: Critical” pathogen in the World Health Organisation list of pathogens for the research, *A. baumannii* imperils the effectiveness of medical treatments for many infectious diseases, even in the most developed countries [32]. This microorganism has, over the years, acquired resistance to most currently available antibiotics, disinfectants, and antiseptics, tolerance to detergents, ultraviolet radiation, desiccation, and the ability to form biofilms on various biotic and abiotic surfaces, which necessitates the introduction of alternative methods of controlling resistant bacterial infections [33,34]. One of the potentially attractive alternatives to addressing the spread of antibiotic-resistant strains is the use of bacteriophages, as well as phage-derived antibacterial proteins [34]. The Acinetobacter phages are of interest because of their ability to further our understanding of these important opportunistic pathogens and their possible exploitation as tools to detect and treat pathogens. Because of its high specificity for host bacteria, bacteriophages have become the target of people’s attention and research [35,36].

Moreover, considering that threats of *A. baumannii* to patients in hospitals and the complete removal of pathogens from hospitals are difficult, effective, and fast analytical methods should be developed for rapid pathogen identification, thereby enabling prompt treatment of patients [37]. Once pathogens are successfully detected and identified, precise treatment can be implemented [38]. Some molecular recognition elements have previously been used to detect bacteria. For example, a paper by Mannoor et al. [39] reported the detection of infectious agents via electronic detection based on antimicrobial peptide-functionalized microcapacitive electrode arrays and another method reported by Miranda et al. [40] was to use a colorimetric enzyme-nanoparticle conjugate system which is featuring quaternary amine headgroups for detection of microbial contamination. The molecular recognition elements show outstanding merits such as low cost and high stability, however, their application is greatly limited by the poor specificity for recognizing given bacteria. Phages have been proposed as alternative agents to detect and treat infectious diseases caused by antibiotic-resistant pathogens [41,42], especially tail fiber proteins derived from bacteriophages. Previous studies have used phages in the preparations of genetically engineered cocktails according to receptor-binding proteins, generally TFP, with a wide range of recognition capabilities [8,43]. Many people also make use of gene-editing technology to artificially modify TFPs to detect and treat pathogenic bacteria [44,45,46,47]. Tail fiber proteins on the tail fibers of bacteriophages can be massively generated through genetic engineering and can be used as suitable probes to target their host bacteria. Recently, Bai et al. described a method for detecting bacteria using TFPs of phages [37]. The authors used the TFPs-magnetic nanoparticles as affinity probes to trap trace *A. baumannii* and used matrix-assisted laser desorption/ionization mass spectrometry as the detection tool.

As a reference, the approach described herein also focuses on using the bacteriophage TFPs to develop an easy method for bacterial detection. In our study, a new bacteriophage obtained from sewage specifically infecting *A. baumannii* was isolated and characterized. Most Acinetobacter phages are tailed viruses with dsDNA genomes which are classified into one of three families of the order of *Caudovirales*; *Myoviridae*, *Podoviridae*, and *Siphoviridae* [48]. Morphologically, AbTJ exhibits features similar to the *Podoviridae* family, which are, generally, tailed phages with icosahedral head symmetry and short tail structures. The adsorption rate and one-step growth curve show that this phage has an extremely long latent period and small burst size. These results are the parameters for determining the efficacy of infective phages on host bacteria and suggestive of AbTJ’s lytic nature.

In order to evaluate the safety of the application of AbTJ at the genetic level, the whole genome was sequenced and analyzed to determine whether the phage genome contained virulence factors and virulence-transferring genes. The results showed that none of the 62 predicted genes expressed in the phage genome contained the above genes, which fully demonstrated its application safety. The phage genome was shown to be dsDNA and showed sequence homology to *Acinetobacter* phage Ab105- 1phi. We believe that combined with the bioinformatics and gene function analyses already performed, further analysis of this phage genome will provide greater information on *Acinetobacter* phages. Phage tail fiber proteins have been regarded as a novel kind of substance for their ability to detect pathogenic bacteria. Previous studies on phage infections have shown that the progression of infection starts when phages interact precisely with receptor molecules on the surface of the host cell [49,50]. Many phages contain specific receptor binding domains, most of which turn out to be tail fiber proteins [10]. As shown in this study, the predicted proteins encoded by ORF52 and ORF53 have been identified as tail fiber proteins. These two TFPs have proven to be receptor binding proteins which have higher flexibility and greater potential for use in detection applications compared to the intact phages. One-site BL and sandwich FL methods were successfully established using gp52 and gp53, with the results proving them to be feasible for use in the detection of *A. baumannii*. A method combining magnetic beads and bioluminescence was used to detect bacteria in complicated sample mixtures, with a recovery rate above 50%. Compared with other rapid detection methods, this method was simple, specific, and sensitive, traits which could significantly improve bacterial detection specificity and efficiency. The study of AbTJ is in its infancy and many questions remain to be answered regarding its TFPs and evolutionary strategies. Future research could focus on broadening the application of these phage tail fiber proteins in the detection of bacteria through biotechnology. Finally, the speed and scale at which phages can be engineered and screened should enable rapid adaptation and discovery of effective antimicrobial agents against the bacterial pathogens.

## Figures and Tables

**Table 1:** Host range infection of the phage AbTJ. −absent; +present.

Species	ID	Infection
*Acinetobacter baumannii*	MDR-TJ(CP003500)	+
*Acinetobacter baumannii*	MDR-A	+
*Acinetobacter baumannii*	MDR-B	+
*Acinetobacter baumannii*	MDR-C	+
*Acinetobacter baumannii*	ATCC19606	+
*Acinetobacter haemolyticus*	TJS01	−
*Acinetobacter haemolyticus*	TJR01	−
*Acinetobacter haemolyticus*	H2063	−
*Acinetobacter haemolyticus*	W65	−
*Bacillus subtilis*	Sck6	−
*Staphylococcus aureus*	MRS-TJ	−
*Escherichia coli*	ATCC25922	−
*Escherichia coli*	MG1655	−
*Escherichia coli*	BL21	−
*Escherichia coli*	DH5α	−
*Escherichia coli*	Rosetta	−
*Escherichia coli*	TG1	−
*Escherichia coli*	TOP10	−
*Escherichia coli*	TransB	−
*Pichia pastoris*	GS115	−

**Table 2:** *Acinetobacter* phage AbTJ gene annotations.

ORFs	Strand	Start	End	Length (aa) ^a^	aa Identity (%)	Function
Orf01	+	179	1441	420	24	Integrase (Pseudomonas phage vB PaeS PMG1)
Orf02	−	1447	1716	89	45	hypothetical protein (Acinetobacter phage Bphi-B1251)
Orf03	−	1717	1974	85	80	hypothetical protein (Acinetobacter phage Ab105-1phi)
Orf04	−	1978	2262	94	37	hypothetical protein
Orf05	−	2259	2468	69	99	hypothetical protein (Acinetobacter phage Ab105-1phi)
Orf06	−	2459	2716	85	92	hypothetical protein (Acinetobacter phage Bphi-B1251)
Orf07	−	2718	3719	333	51	hypothetical protein (Acinetobacter phage Bphi-B1251)
Orf08	−	3716	4837	373	94	phage nucleotide-binding protein (Acinetobacter phage Bphi-B1251)
Orf09	−	4848	5171	107	93	hypothetical protein (Acinetobacter phage Bphi-B1251)
Orf10	−	5174	5614	156	96	hypothetical protein (Acinetobacter phage Ab105-1phi)
Orf11	−	5843	6634	263	26	putative transcriptional regulator (Acinetobacter phage Ab105-1phi)
Orf12	+	6637	6885	82	45	regulatory protein cro (Escherichia phage HK75)
Orf13	+	6940	7440	166	38	hypothetical protein (Acinetobacter phage Bphi-B1251)
Orf14	+	7490	7762	90	51	hypothetical protein (Acinetobacter phage Ab105-1phi)
Orf15	+	7759	8055	98	98	hypothetical protein (Acinetobacter phage Ab105-1phi)
Orf16	+	8052	8408	80	97	hypothetical protein (Acinetobacter phage Ab105-1phi)
Orf17	+	8408	9337	309	36	hypothetical protein BA3 0036[Thalassomonas phage BA3)
Orf18	+	9330	10,079	249	—	hypothetical protein
Orf19	+	10,076	10,498	140	99	hypothetical protein (Acinetobacter phage Bphi-B1251)
Orf20	+	10,488	10,910	140	99	hypothetical protein (Acinetobacter phage Ab105-1phi)
Orf21	+	10,903	11,127	75	74	hypothetical protein (Acinetobacter phage Bphi-B1251)
Orf22	+	11,127	11,522	131	94	hypothetical protein (Acinetobacter phage Bphi-B1251)
Orf23	+	11,519	12,019	166	99	hypothetical protein (Acinetobacter phage Ab105-1phi)
Orf24	+	12,215	12,865	216	99	hypothetical protein (Acinetobacter phage Ab105-1phi)
Orf25	+	13,127	13,885	252	—	hypothetical protein
Orf26	+	13,980	14,627	215	—	hypothetical protein
Orf27	+	14,675	15,184	169	44	hypothetical protein (Pectobacterium phage ZF40)
Orf28	+	15,181	16,839	552	40	terminase large subunit (Burkholderia phage BcepB1A)
Orf29	+	16,850	18,262	470	32	putative portal protein (Vibrio phage CP-T1)
Orf30	−	18,060	17,848	70	21	hypothetical protein (Psychrobacter phage pOW20-A)
Orf31	+	19,209	20,525	438	—	hypothetical protein
Orf32	+	20,529	21,005	158	89	hypothetical protein (Acinetobacter phage Ab105-1phi)
Orf33	+	21,070	22,095	341	—	hypothetical protein
Orf34	+	22,105	22,536	143	—	hypothetical protein
Orf35	+	22,540	22,926	128	99	hypothetical protein
Orf36	+	22,923	23,483	186	30	hypothetical protein (Klebsiella phage JD001)
Orf37	+	23,515	23,838	107	99	hypothetical protein (Acinetobacter phage Ab105-1phi)
Orf38	+	23,841	24,380	179	42	hypothetical protein (Aggregatibacter phage S1249)
Orf39	+	24,384	25,859	491	99	hypothetical protein (Acinetobacter phage Ab105-1phi)
Orf40	+	25,874	26,317	147	99	hypothetical protein
Orf41	+	26,317	26,781	154	66	hypothetical protein (Acinetobacter phage Ab105-1phi)
Orf42	+	26,911	29,001	696	99	hypothetical protein (Acinetobacter phage Ab105-1phi)
Orf43	−	28,998	29,354	118	98	hypothetical protein (Acinetobacter phage Ab105-1phi)
Orf44	+	29,586	29,774	62	99	hypothetical protein (Acinetobacter phage Ab105-1phi)
Orf45	+	29,941	30,537	198	27	hypothetical protein (Psychrobacter phage pOW20-A)
Orf46	+	30,540	30,836	98	34	hypothetical protein (Psychrobacter phage pOW20-A)
Orf47	+	30,833	31,792	319	26	hypothetical protein (Aggregatibacter phage S1249)
Orf48	+	31,795	32,457	220	35	hypothetical protein (Psychrobacter phage pOW20-A)
Orf49	+	32,492	32,845	117	99	hypothetical protein (Acinetobacter phage Ab105-1phi)
Orf50	+	32,848	34,032	394	99	hypothetical protein (Acinetobacter phage Ab105-1phi)
Orf51	+	34,032	34,622	196	93	hypothetical protein (Acinetobacter phage Ab105-1phi)
Orf52	+	34,615	35,223	202	99	tail fiber protein (Acinetobacter phage Ab105-1phi)
Orf53	+	35,242	37,341	699	96	tail fiber protein (Acinetobacter phage Ab105-1phi)
Orf54	+	37,343	37,570	75	96	hypothetical protein (Acinetobacter phage Ab105-1phi)
Orf55	+	37,648	38,037	129	100	hypothetical protein (Acinetobacter phage Ab105-1phi)
Orf56	+	38,080	38,625	181	80	secretion activator protein (Acinetobacter phage Ab105-1phi)
Orf57	+	38,814	39,494	226	27	hypothetical protein (Acinetobacter phage Ab105-1phi)
Orf58	+	39,505	40,152	215	98	hypothetical protein (Acinetobacter phage Bphi-B1251)
Orf59	+	40,159	40,752	197	62	hypothetical protein (Acinetobacter phage Bphi-B1251)
Orf60	−	40,757	40,906	50	99	lesion bypass DNA polymerase V (Acinetobacter phage Ab105-1phi)
Orf61	−	40,998	41,852	284	14	Transposase (Acinetobacter phage Ab105-1phi)
Orf62	−	41,864	42,163	99	27	transposase (Acinetobacter baumannii OIFC099)

^a^ Amino acids.

**Table 3:** Results of the test bacterial concentration and recovery tests of *A. baumanni* spread in real samples by the gp52-MBs with the observed bioluminescence signal value (*n* = 4).

Sample	Test Bacterial Concentration (CFU mL^−1^)	Bioluminescence Signal Value (a.u)	Bacteria Captured on the Beads (CFU mL^−1^)	Recovery (%)	RSD (%)
Glucose injection	1.5 × 10^6^	1094.1	1.34 × 10^6^	89.3	5.6
	1.5 × 10^5^	505.8	1.28 × 10^5^	85.3	1.5
	1.5 × 10^4^	222.3	1.11 × 10^4^	74.0	3.1
	1.5 × 10^3^	95.7	8.58 × 10^2^	57.2	3.8
Human urine	1.5 × 10^6^	1100.4	1.34 × 10^6^	89.3	5.3
	1.5 × 10^5^	495.5	1.25 × 10^5^	83.3	3.1
	1.5 × 10^4^	218.3	1.05 × 10^4^	70.0	2.7
	1.5 × 10^3^	92.9	8.44 × 10^2^	56.3	0.1
Human feces	1.5 × 10^6^	1076.5	1.26 × 10^6^	84	5.8
	1.5 × 10^5^	486.4	1.14 × 10^5^	76	1.9
	1.5 × 10^4^	217.3	1.02 × 10^4^	68.0	3.1
	1.5 × 10^3^	92.3	8.77 × 10^2^	58.2	3.1
Human sputum	1.5 × 10^6^	1090.6	1.31 × 10^6^	87.3	1.4
	1.5 × 10^5^	489.8	1.17 × 10^5^	78.0	2.3
	1.5 × 10^4^	214.3	9.80 × 10^3^	65.3	1.7
	1.5 × 10^3^	91.8	8.22 × 10^2^	54.8	3.8
Domestic sewage	1.5 × 10^6^	1071.2	1.24 × 10^6^	82.7	1.4
	1.5 × 10^5^	483.1	1.12 × 10^5^	76.7	1.9
	1.5 × 10^4^	211.3	9.21 × 10^3^	61.4	3.1
	1.5 × 10^3^	90.3	7.67 × 10^2^	51.1	3.0

**Table 4:** Results of the test bacterial concentration and recovery tests of *A. baumanni* spread in real samples by the gp53-MBs with the observed bioluminescence signal value (*n* = 4).

Sample	Test Bacterial Concentration (CFU mL^−1^)	Bioluminescence Signal Value (a.u)	Bacteria Captured on the Beads (CFU mL^−1^)	Recovery (%)	RSD (%)
Glucose injection	1.5 × 10^6^	1030.3	1.39 × 10^6^	92.7	0.7
	1.5 × 10^5^	465.6	1.34 × 10^5^	89.3	1.8
	1.5 × 10^4^	199.9	1.11 × 10^4^	74	4.0
	1.5 × 10^3^	83.8	8.43 × 10^2^	56.3	1.2
Human urine	1.5 × 10^6^	1025.7	1.39 × 10^6^	92.7	1.0
	1.5 × 10^5^	459.2	1.28 × 10^5^	85.3	1.8
	1.5 × 10^4^	197.2	1.06 × 10^4^	70.7	2.4
	1.5 × 10^3^	80.4	7.83 × 10^2^	52.2	3.6
Human feces	1.5 × 10^6^	1020.9	1.37 × 10^6^	91.3	0.9
	1.5 × 10^5^	454.9	1.28 × 10^5^	85.3	2.5
	1.5 × 10^4^	195.9	1.04 × 10^4^	69.3	2.4
	1.5 × 10^3^	80.1	7.74 × 10^2^	51.6	3.6
Human sputum	1.5 × 10^6^	1018.7	1.33 × 10^6^	90.6	1.5
	1.5 × 10^5^	456.0	1.27 × 10^5^	84.7	1.5
	1.5 × 10^4^	195.4	1.04 × 10^4^	69.3	2.8
	1.5 × 10^3^	80.1	7.74 × 10^2^	51.6	4.4
Domestic sewage	1.5 × 10^6^	1016.2	1.32 × 10^6^	88.0	1.5
	1.5 × 10^5^	450.8	1.22 × 10^5^	81.3	1.4
	1.5 × 10^4^	192.3	9.94 × 10^3^	66.7	2.9
	1.5 × 10^3^	79.3	7.57 × 10^2^	50.5	4.3

## Supplementary Materials

The following are available online at https://www.mdpi.com/1999-4915/12/2/205/s1, Figure S1: Restriction fragment length polymorphism analysis of AbTJ DNA.

## References

[1] Dijkshoorn L., Nemec A., Seifert H. (2007). An increasing threat in hospitals: Multidrug-resistant Acinetobacter baumannii. Nat. Rev. Microbiol..

[2] Dubrovin E.V., Popova A.V., Kraevskiy S.V., Ignatov S.G., Ignatyuk T.E., Yaminsky I.V., Volozhantsev N.V. (2012). Atomic force microscopy analysis of the Acinetobacter baumannii bacteriophage AP22 lytic cycle. PLoS ONE.

[3] Jacobs A.C., Thompson M.G., Gebhardt M., Corey B.W., Yildirim S., Shuman H.A., Zurawski D.V. (2014). Genetic Manipulation of Acinetobacter baumannii. Curr. Protoc. Microbiol..

[4] He Y., Shi Y., Liu M., Wang Y., Wang L., Lu S., Fu Z. (2018). Nonlytic Recombinant Phage Tail Fiber Protein for Specific Recognition of Pseudomonas aeruginosa. Anal. Chem..

[5] Kazaks A., Dislers A., Lipowsky G., Nikolajeva V., Tars K. (2012). Complete genome sequence of the Enterobacter cancerogenus bacteriophage Enc34. J. Virol..

[6] Cui Z., Shen W., Wang Z., Zhang H., Me R., Wang Y., Zeng L., Zhu Y., Qin J., He P. (2012). Complete genome sequence of Klebsiella pneumoniae phage JD001. J. Virol..

[7] Krasowska A., Biegalska A., Augustyniak D., Los M., Richert M., Lukaszewicz M. (2015). Isolation and Characterization of Phages Infecting Bacillus subtilis. Biomed. Res. Int..

[8] Wiggins B.A., Alexander M. (1985). Minimum bacterial density for bacteriophage replication: Implications for significance of bacteriophages in natural ecosystems. Appl. Environ. Microbiol..

[9] Prevelige P.E., Cortines J.R. (2018). Phage assembly and the special role of the portal protein. Curr. Opin. Virol..

[10] Nobrega F.L., Vlot M., de Jonge P.A., Dreesens L.L., Beaumont H.J.E., Lavigne R., Dutilh B.E., Brouns S.J.J. (2018). Targeting mechanisms of tailed bacteriophages. Nat. Rev. Microbiol..

[11] Wu L., Huang T., Yang L., Pan J., Zhu S., Yan X. (2011). Sensitive and selective bacterial detection using tetracysteine-tagged phages in conjunction with biarsenical dye. Angew. Chem. Int. Ed. Engl..

[12] Chen J., Alcaine S.D., Jackson A.A., Rotello V.M., Nugen S.R. (2017). Development of Engineered Bacteriophages for Escherichia coli Detection and High-Throughput Antibiotic Resistance Determination. ACS Sens..

[13] Huang H., Yang Z.L., Wu X.M., Wang Y., Liu Y.J., Luo H., Lv X., Gan Y.R., Song S.D., Gao F. (2012). Complete genome sequence of Acinetobacter baumannii MDR-TJ and insights into its mechanism of antibiotic resistance. J. Antimicrob. Chemother..

[14] Gao F., Wang Y., Liu Y.J., Wu X.M., Lv X., Gan Y.R., Song S.D., Huang H. (2011). Genome sequence of Acinetobacter baumannii MDR-TJ. J. Bacteriol..

[15] Huang H., Dong Y., Yang Z.L., Luo H., Zhang X., Gao F. (2014). Complete Sequence of pABTJ2, A Plasmid from Acinetobacter baumannii MDR-TJ, Carrying Many Phage-like Elements. Genom. Proteom. Bioinform..

[16] Luo J., Jiang M., Xiong J., Li J., Zhang X., Wei H., Yu J. (2018). Exploring a phage-based real-time PCR assay for diagnosing Acinetobacter baumannii bloodstream infections with high sensitivity. Anal. Chim. Acta.

[17] Hua Y., Luo T., Yang Y., Dong D., Wang R., Wang Y., Xu M., Guo X., Hu F., He P. (2017). Phage Therapy as a Promising New Treatment for Lung Infection Caused by Carbapenem-Resistant Acinetobacter baumannii in Mice. Front. Microbiol..

[18] Mendes J.J., Leandro C., Mottola C., Barbosa R., Silva F.A., Oliveira M., Vilela C.L., Melo-Cristino J., Gorski A., Pimentel M. (2014). In vitro design of a novel lytic bacteriophage cocktail with therapeutic potential against organisms causing diabetic foot infections. J. Med. Microbiol..

[19] Li Y., Wang M., Liu Q., Song X., Wang D., Ma Y., Shao H., Jiang Y. (2016). Complete Genomic Sequence of Bacteriophage H188: A Novel Vibrio kanaloae Phage Isolated from Yellow Sea. Curr. Microbiol..

[20] Li E., Yin Z., Ma Y., Li H., Lin W., Wei X., Zhao R., Jiang A., Yuan J., Zhao X. (2016). Identification and molecular characterization of bacteriophage phiAxp-2 of Achromobacter xylosoxidans. Sci. Rep..

[21] Jang S.H., Yoon B.H., Chang H.I. (2011). Complete nucleotide sequence of the temperate bacteriophage LBR48, a new member of the family Myoviridae. Arch. Virol..

[22] Gilbert R.A., Kelly W.J., Altermann E., Leahy S.C., Minchin C., Ouwerkerk D., Klieve A.V. (2017). Toward Understanding Phage:Host Interactions in the Rumen; Complete Genome Sequences of Lytic Phages Infecting Rumen Bacteria. Front. Microbiol..

[23] Tang L.H., Jian H.H., Song C.Y., Bao D.P., Shang X.D., Wu D.Q., Tan Q., Zhang X.H. (2013). Transcriptome analysis of candidate genes and signaling pathways associated with light-induced brown film formation in Lentinula edodes. Appl. Microbiol. Biotechnol..

[24] Bankevich A., Nurk S., Antipov D., Gurevich A.A., Dvorkin M., Kulikov A.S., Lesin V.M., Nikolenko S.I., Pham S., Prjibelski A.D. (2012). SPAdes: A new genome assembly algorithm and its applications to single-cell sequencing. J. Comput. Biol..

[25] Chen Y., Li X., Song J., Yang D., Liu W., Chen H., Wu B., Qian P. (2019). Isolation and characterization of a novel temperate bacteriophage from gut-associated Escherichia within black soldier fly larvae (Hermetia illucens L. [Diptera: Stratiomyidae]). Arch. Virol..

[26] Carver T., Thomson N., Bleasby A., Berriman M., Parkhill J. (2009). DNAPlotter: Circular and linear interactive genome visualization. Bioinformatics.

[27] Khalil M.A., Azzazy H.M., Attia A.S., Hashem A.G. (2014). A sensitive colorimetric assay for identification of Acinetobacter baumannii using unmodified gold nanoparticles. J. Appl. Microbiol..

[28] Holtappels M., Vrancken K., Schoofs H., Deckers T., Remans T., Noben J.P., Valcke R. (2015). A comparative proteome analysis reveals flagellin, chemotaxis regulated proteins and amylovoran to be involved in virulence differences between Erwinia amylovora strains. J. Proteom..

[29] Ma Y., Li E., Qi Z., Li H., Wei X., Lin W., Zhao R., Jiang A., Yang H., Yin Z. (2016). Isolation and molecular characterisation of Achromobacter phage phiAxp-3, an N4-like bacteriophage. Sci. Rep..

[30] Jeon J., D’Souza R., Pinto N., Ryu C.M., Park J., Yong D., Lee K. (2016). Characterization and complete genome sequence analysis of two Myoviral bacteriophages infecting clinical carbapenem-resistant Acinetobacter baumannii isolates. J. Appl. Microbiol..

[31] Moller-Olsen C., Ho S.F.S., Shukla R.D., Feher T., Sagona A.P. (2018). Engineered K1F bacteriophages kill intracellular Escherichia coli K1 in human epithelial cells. Sci. Rep..

[32] World Health Organisation (2017). Global Priority List of Antibiotic-Resistant Bacteria to Guide Research, Discovery and Development of New Antibiotics.

[33] Peleg A.Y., Seifert H., Paterson D.L. (2008). Acinetobacter baumannii: Emergence of a successful pathogen. Clin. Microbiol. Rev..

[34] Popova A.V., Lavysh D.G., Klimuk E.I., Edelstein M.V., Bogun A.G., Shneider M.M., Goncharov A.E., Leonov S.V., Severinov K.V. (2017). Novel Fri1-like viruses infecting Acinetobacter baumannii—vB_AbaP_AS11 and vB_AbaP_AS12—characterization, comparative genomic analysis, and host-recognition strategy. Viruses.

[35] Turner D., Ackermann H.W., Kropinski A.M., Lavigne R., Sutton J.M., Reynolds D.M. (2018). Comparative analysis of 37 Acinetobacter bacteriophages. Viruses.

[36] Plotka M., Kapusta M., Dorawa S., Kaczorowska A.K., Kaczorowski T. (2019). Ts2631 endolysin from the extremophilic thermus scotoductus bacteriophage vB_Tsc2631 as an antimicrobial agent against gram-negative multidrug-resistant bacteria. Viruses.

[37] Bai Y.L., Shahed-Al-Mahmud M., Selvaprakash K., Lin N.T., Chen Y.C. (2019). Tail Fiber Protein-Immobilized Magnetic Nanoparticle-Based Affinity Approaches for Detection of Acinetobacter baumannii. Anal. Chem..

[38] Lu T.K., Bowers J., Koeris M.S. (2013). Advancing bacteriophage-based microbial diagnostics with synthetic biology. Trends Biotechnol..

[39] Mannoor M.S., Zhang S., Link A.J., McAlpine M.C. (2010). Electrical detection of pathogenic bacteria via immobilized antimicrobial peptides. Proc. Natl. Acad. Sci. USA.

[40] Miranda O.R., Li X.N., Garcia-Gonzalez L., Zhu Z.J., Yan B., Bunz U.H.F., Rotello V.M. (2011). Colorimetric bacteria sensing using a supramolecular enzyme-nanoparticle biosensor. J. Am. Chem. Soc..

[41] Saussereau E., Debarbieux L. (2012). Bacteriophages in the experimental treatment of Pseudomonas aeruginosa infections in mice. Adv. Virus Res..

[42] Jiang L., Schlesinger F., Davis C.A., Zhang Y., Li R., Salit M., Gingeras T.R., Oliver B. (2011). Synthetic spike-in standards for RNA-seq experiments. Genome Res..

[43] Mahichi F., Synnott A.J., Yamamichi K., Osada T., Tanji Y. (2009). Site-specific recombination of T2 phage using IP008 long tail fiber genes provides a targeted method for expanding host range while retaining lytic activity. FEMS Microbiol. Lett..

[44] Monteiro R., Pires D.P., Costa A.R., Azeredo J. (2019). Phage Therapy: Going Temperate?. Trends Microbiol..

[45] Hatoum-Aslan A. (2018). Phage Genetic Engineering Using CRISPR(-)Cas Systems. Viruses.

[46] Pires D.P., Cleto S., Sillankorva S., Azeredo J., Lu T.K. (2016). Genetically Engineered Phages: A Review of Advances over the Last Decade. Microbiol. Mol. Biol. Rev..

[47] Fernandes S., Sao-Jose C. (2018). Enzymes and Mechanisms Employed by Tailed Bacteriophages to Breach the Bacterial Cell Barriers. Viruses.

[48] Park J.Y., Kim S., Kim S.M., Cha S.H., Lim S.K., Kim J. (2011). Complete genome sequence of multidrug-resistant Acinetobacter baumannii strain 1656-2, which forms sturdy biofilm. J. Bacteriol..

[49] Higgins P.G., Dammhayn C., Hackel M., Seifert H. (2010). Global spread of carbapenem-resistant Acinetobacter baumannii. J. Antimicrob. Chemother..

[50] Tao P., Wu X., Tang W.C., Zhu J., Rao V. (2017). Engineering of Bacteriophage T4 Genome Using CRISPR-Cas9. ACS Synth. Biol..

