# Supplementary material for: Isolation and Characterization of AbTJ, an Acinetobacter baumannii Phage, and Functional Identification of Its Receptor-Binding Modules
Source: Viruses. 2020 Feb 12;12(2):205. doi: 10.3390/v12020205 (PMC7077233; doi:10.3390/v12020205)
Supplement: Supplementary file 1 [file viruses-12-00205-s001.pdf]

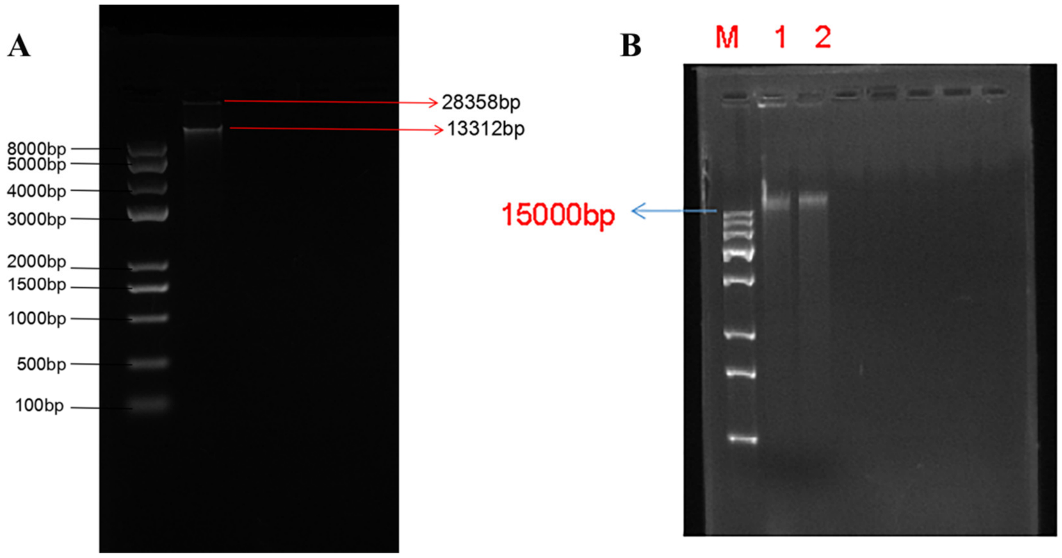


Figure 1. Restriction fragment length polymorphism analysis of AbTJ DNA. Genomic DNA from phage AbTJ was digested with the enzymes indicated (SacII and DrdI) and run on an agarose gel (0.8%).
